# Detection of pesticide residues and risk assessment from the local fruits and vegetables in Incheon, Korea

**DOI:** 10.1038/s41598-022-13576-5

**Published:** 2022-06-10

**Authors:** Byung Kyu Park, Sung Hee Kwon, Mi Sook Yeom, Kwang Sig Joo, Myung Je Heo

**Affiliations:** 1 Samsan Agricultural Products Inspection Center, Incheon Institute of Public Health and Environment, 46, Yeongseongdong-ro, Bupyeong-gu, Incheon, 21320 Republic of Korea; 2 Department of Food Science and Biotechnology, Sejong University, 209, Neungdong-ro, Gwangjin-gu, Seoul, 05006 Republic of Korea

**Keywords:** Environmental sciences, Risk factors, Chemistry

## Abstract

This study was conducted to investigate the pesticide residue concentrations and assess potential human health risks from fruit and vegetable consumption in Incheon. A total of 1,146 samples of 20 different types of fruits and vegetables were collected from the Incheon area in 2020. The pesticide residues were analyzed by the multi-residue method of the Korean Food Code for 400 different pesticides. Among the fruit and vegetable samples, 1,055 samples (92.1%) were free from detectable residues, while 91 samples (7.9%) contained residues and 11 samples (1.0%) had residues exceeding the Korean maximum residue limit. A total of 32 different pesticide residues were found and 8 residues exceeded MRLs. The most frequently detected pesticide residues were chlorfenapyr, procymidone, etofenprox, pendimethalin, fluopyram and azoxystrobin. The highest values of short term and long term exposure were obtained in the case of consumption of lettuce(leaves) with chlorfenpyr. For chronic dietary exposure, the cumulative hazard index (cHI) were below 100%. The results of this study showed that the detected pesticides were not exposed to potential health risks through the consumption of fruits and vegetables.

## Introduction

Pesticides are essential tools to increase agricultural productivity and cultivation convenience by protecting crops from pests pathogen and weeds. However, pesticides inevitably remain in agricultural products and soil^1,2^. Excessive use of pesticides causes these chemicals and metabolites to remain in the environment and food, causing serious problems in the ecosystem and public health^3–5^. Chronic human exposure to unsafe levels of pesticides can cause a wide range of diseases affecting human health. Pesticides have potential adverse effects on human health such as carcinogenesis, immunotoxicity, birth defects, genetic changes, neurological toxicity and endocrine disruption^6–8^. Fruits and vegetables are usually consumed directly without processing after washing, so they are the main cause of pesticide residue ingestion in humans. Human intakes of hazardous substances from pesticide residues in agricultural products can be significantly higher than intakes of these substances associated with water consumption and air intake^9,10^. Therefore, it is very important to monitor residual pesticides in fruits and vegetables and to assess if they cause a risk to human health.

Fruits and vegetables are essential for human nutrition containing functional compounds such as carotenoids, phenolics, trace minerals, vitamins and fiber^11^. However, they may contain toxic residual pesticides due to the use of pesticides during the production process of agricultural products^12^. Pesticide residues in agricultural products are usually monitored with reference to maximum residue limits (MRLs), which represent the highest concentration of pesticide residues that is legally permitted or accepted in food commodities after the use of pesticides^13,14^. In Korea, the Ministry of Food and Drug Safety and the Rural Development Administration are responsible for managing pesticides. To ensure the safety of agricultural products, Korea has implemented the PLS (Positive List System) since 2019. PLS register pesticides used in domestic or imported foods and establishes pesticide MRLs in agricultural products. It is a system that pesticide MRLs in agricultural products is applied at 0.01 mg/kg uniformly except for registered pesticides^15^. Government and related organizations are trying to ensure safe use of pesticides, but pesticide residues are continuously detected in agricultural products. Some agricultural products occur in excess of MRLs and exposure to pesticide residues is likely to harm humans. The purpose of this study was to measure the concentration of pesticide residues in fruits and vegetables and evaluate whether these residues pose a risk to the consumer’s health.

## Materials and methods

### Chemical and reagents

In this study, tests were conducted on 400 pesticides that can be analyzed by the multi-residue method of the Korean Food Code. The 400 pesticide standards used to analyze pesticide residues were purchased by Accustandard (New Haven, CT, USA). For the extraction of pesticide residues, acetonitrile, acetone, dichloromethane and *n*-hexane used in this experiment were purchase with HPLC grade reagents (Muskegon, MI, USA) and anhydrous sodium chloride was purchased from Junsei (Tokyo, Japan). Solid phase extraction (SPE) for sample purification was purchased from Bekolut (Haupststuhl, Rhineland-Palatinate, Germany).

### Samples

Sampling was performed by an authorized person who met the Korean Food Code Guideline. Samples used in this experiment were collected from 1146 fruits and vegetables from markets in Incheon, Republic of Korea in 2020. The samples were collected from fresh agricultural products and quickly brought to the laboratory for analysis. All samples were analyzed within 24 h and kept in the refrigerator until extraction.

### Sample extraction and clean up

Sample extraction and clean up were performed according to the multi-residue method for pesticide residues according to the Korean Food Code. Part of the collected samples (1 kg) were taken and thoroughly crushed with a food grinder. After grinding, 100 mL acetonitrile was added to the ground sample and homogenized for 3 min with a high-speed homogenizer. The homogenized mixture was filtered into a bottle with 15 g of anhydrous sodium chloride and mix filtrate vigorously for 3 min to separate layers. From the upper layer, an aliquot of 20 mL was transferred into a tube and evaporated to dryness on a 40 °C bath with a gentle stream of air. For GC–MS/MS and GC-ECD/NPD analysis, the dried extracts were dissolved with 4 mL of acetone/*n*-hexane (20:80, v/v) and transferred to a Florisil cartridge (1 g, 6 mL), which was activated and pre-conditioned with 5 mL of acetone/*n*-hexane (20:80, v/v) and 5 mL of *n*-hexane. After sample loading, the cartridge was eluted with 5 mL of acetone/*n*-hexane (20:80, v/v). This solvent was then evaporated slowly to dryness at 40 °C bath under a gentle stream of air. The dried residue was re-dissolved with 2 mL of acetone/*n*-hexane (20:80, v/v) and filtered with 0.2 µm PTFE filter (Advantec, Otowa, Tokyo, Japan) for GC analysis. For LC–MS/MS and LC-UVD analysis, sample extracts were dissolved with 4 mL of dichloromethane/methanol (99:1, v/v) and transferred to amino-propyl cartridge (1 g, 6 mL) which was activated and pre-conditioned with 5 mL of dichloromethane. After sample loading, the cartridge was eluted with 7 mL of dichloromethane/methanol (99:1, v/v). This solvent was evaporated slowly to dryness at 40 °C bath under a gentle stream of air. The dried residue was re-dissolved with 2 mL of acetonitrile and filtered with 0.2 µm PTFE filter for LC analysis.

### GC–MS/MS analysis

GC–MS/MS analysis was performed using 7890B gas chromatograph coupled to a triple quadrupole mass spectrometer 7000D with electron impact ionization (EI) equipped with a 7693 autosampler (Agilent Technologies, Santa Clara, CA, USA). Chromatographic separation of pesticides was conducted on DB-5 MS capillary columns (30 m × 0.25 mm × 0.25 µm film thickness, Agilent Technologies, Santa Clara, CA, USA). The oven temperature was programmed from 70 °C (hold 3 min) to 180 °C by a rate of 20 °C/min and finally increased to 300 °C (hold 2.5 min) by a rate of 5 °C/min. The temperature of injector was held at 250 °C and the injection volume was 1 µL with splitless mode. Helium carrier gas (99.999%) flowed constantly at 1 mL/min. The mass spectrometry detector (MSD) used electron impact ionization mode (ionization energy 70 eV). The temperature of ion source and quadrupole were set at 250 °C and 150 °C, respectively. The multiple reaction monitoring (MRM) mode with minimum two ions for each pesticide was used for detection and quantification of pesticides.

### GC-ECD/NPD analysis

The GC-ECD and GC-NPD system was used to analyze organochlorine and pyrethroid compounds, organophosphorus and nitrogen-containing compounds. An Agilent 6890 series GC equipped with ^63^Ni electron capture detector and a nitrogen phosphorous detector were employed. Chromatographic separations were conducted on DB-5 capillary columns (30 m × 0.25 mm × 0.25 µm film thickness, Agilent Technologies, Santa Clara, CA, USA) for GC-ECD and GC-NPD. The operating conditions for GC-ECD were as follows: The oven temperature was programmed from 150 °C (hold 1 min) to 240 °C (hold 2 min) by a rate of 12 °C/min and finally increased to 280 °C (hold 13.5 min) by a rate of 10 °C/min. The injection volume was 1µL with split mode (42.2:1) and nitrogen carrier gas flowed at 1.2 mL/min. The temperature of injector and detector were at 250 °C and 280 °C, respectively. The operating conditions for GC-NPD were as follows : The oven temperature was programmed from 120 °C (hold 1 min) to 240 °C (hold 2 min) by a rate of 12 °C/min, increased to 280 °C (hold 10 min) by a rate of 10 °C/min and finally increased to 300 °C (hold 1 min) by a rate of 10 °C/min. The injection volume was 1µL with splitless mode. Nitrogen carrier, hydrogen and air flowed at 1.2 mL/min, at 3.0 mL/min, at 120.0 mL/min, respectively. The temperature of injector and detector were at 270 °C and 300 °C, respectively.

### LC–MS/MS analysis

LC–MS/MS analysis was performed using Vanquish UHPLC system coupled to a TSQ Altis triple quadrupole mass detector system (Thermo-Fisher Scientific, Waltham, Massachusetts, USA). Chromatographic separation of pesticides was performed using Accucore aQ (2.1 mm × 100 mm, 2.6 µm particle size, Thermo-Fisher Scientific, Waltham, Massachusetts, USA). Mobile phase A (0.01% formic acid and 5 mM ammonium formate in water) and mobile phase B (0.01% formic acid and 5 mM ammonium formate in methanol) were used for the gradient program. The gradient program was as follows: 0–0.5 min (80% A/20% B), 0.5–12 min (20–95% B), 12–12.1 min (5–80% A) and 12.1–15 min (80% A/20% B). The injection volume was 2 µL with a constant flow rate of 0.3 mL/min and 40 °C oven temperature. For mass spectrometric analysis, LC-MSD was performed using an electrospray ionization source (ESI) in positive and negative modes and data were acquired in SRM mode. Operating conditions were as follows: 350 °C vaporizer temperature, 325 °C ion transfer tube temperature, 3800 eV ion spray voltage, 4.58 L/min sheath gas flow rate. Collision induced dissociation was performed using argon as the collision gas pressure at a 1.5 mTorr in the collision tube.

### HPLC analysis

The high performance liquid chromatography (HPLC) was carried out on Ultimate 3000 (Dionex, Sunnyvale, California, USA) with UV-VWD detector. Chromatographic separation was performed on a Capcell Core C18 column (4.6 mm × 100 mm, 2.7 µm particle size, Osaka Soda, Osaka, Japan). HPLC conditions consisted of mobile phase A (5% acetonitrile in water), mobile phase B (20% methanol/80% acetonitrile, v/v), 10 µL injection volume, 1.0 mL/min flow rate and 40 °C oven temperature. UV absorbance was monitored at 220 nm and 250 nm. The gradient program was as follows: initial (90% A/10% B), 0–13 min (10–80% B), 13–16 min (20% A), 16–16.1 min (20–90% A) and 16.1–20 min (90% A/10% B).

### Method validation

The analytical methods was validated in terms of limit of detection (LOD), limit of quantification (LOQ), recovery and precision according to the Korea Food Code pesticides guidelines^16^. Assessment of recovery was performed using a mixture of the targeted pesticides at fortification levels of 0.1, 1.0 mg/kg using pesticide free sample extracts. The LOD and LOQ were estimated from the standard deviation of the five replicated analyses of spiked sample at low concentration level (LOD = 3.3 × SD and LOQ = 10 × SD). Precision was expressed the relative standard deviation (RSD, %) and was evaluated by analyzing replicate samples. To assess linearity, the extracts from pesticide free samples were fortified with standard solutions of 0.05, 0.1, 0.25, 1.0, 2.0 mg/kg and analyzed in triplicate at each concentration.

### Risk assessment estimation

The risk assessment of pesticide detected in fruits and vegetables was estimated based on the results of the survey on residual pesticides. The short term risk assessment (aHQ) was calculated based on the estimated short term intake (ESTI) and the acute reference dose (ARfD). ESTI was calculated by multiplying the highest residue level and food consumption and dividing this by the body weight. The aHQ was calculated using the formula: aHQ = ESTI/ARfD × 100%. The long term risk assessment (HQ) was performed using the estimated daily intake (EDI) and the established acceptable daily intake (ADI). EDI was calculated by multiplying the average pesticide concentration and the food consumption rate and dividing this by the body weight^17^. The HQ was calculated using the formula: HQ = EDI/ADI × 100%. The average daily intake was referred to the intake of fruits and vegetables examined by Korea Disease Control and Prevention Agency^18^. The criteria for ADI and ARfD refer to pesticides and veterinary drugs information from Ministry of Food and Drug Safety^19^. A value below 100% indicated that the exposed people were unlikely to experience obvious adverse effects. An index above 100% indicated the possibility that the exposure would induce obvious adverse effects^20^. HQ was calculated for the pesticides and agricultural products. The results were summed up to obtain a chronic hazard index (cHI). The chronic hazard index was calculated by the sum of HQs (cHI = ΣHQ). A cHI (%) > 100 indicated that the fruits and vegetables should be considered a risk to the consumers, whereas an index below 100 indicated that the consumption of the fruits and vegetables was considered acceptable^17^.

### Approvals and permissions

This study was approved by Incheon Metropolitan Government for permission to collect agricultural products/plants specimens.

## Results and discussion

### Method validation

From 400 pesticides, 15 pesticides were selected considering the detection rate and the violation rate of the MRLs. Table 1 presents linear correlation coefficients, limits of detection (LODs), limits of quantification (LOQs) and recoveries for the validation study. A linear correlation coefficient between pesticide concentrations and peak areas was detected in the range of 0.9947–0.9999. The LODs and LOQs values for the studied pesticides ranged from 0.004 to 0.040 mg/kg and from 0.011 to 0.120 mg/kg, respectively. The recovery was 85.3–98.3% for all pesticides, which is within the acceptable recovery range of 70–120% and the RSD of less than 10% also met requirement^21^. These results indicate that the analytical method applied to this study is appropriate for the analysis of targeted pesticide residues in fruits and vegetables.

**Table 1:** Validation parameters of the analytical method for pesticide residues detected in this study.

Pesticide	R^2^	LOD (mg/kg)	LOQ (mg/kg)	Recovery (%)	RSD (± , %)
Azoxystrobin	0.9982	0.013	0.041	85.3	4.3
Chlorfenapyr	0.9947	0.012	0.035	98.3	3.5
Etofenprox	0.9963	0.005	0.015	97.8	2.9
Fenobucarb	0.9998	0.017	0.051	95.3	3.2
Flubendiamide	0.9986	0.012	0.035	88.3	2.6
Fludioxonil	0.9995	0.023	0.071	89.7	5.3
Fluopyram	0.9993	0.022	0.067	92.6	4.8
Fluquinconazole	0.9999	0.009	0.027	96.4	2.5
Hexaconazole	0.9967	0.040	0.120	86.1	6.4
Methidathion	0.9997	0.007	0.021	92.4	2.1
Pendimethalin	0.9978	0.004	0.012	95.6	1.3
Prochloraz	0.9997	0.013	0.038	90.9	5.9
Procymidone	0.9988	0.014	0.043	89.7	5.2
Tebuconazole	0.9996	0.004	0.011	91.1	0.5
Tebufenpyrad	0.9990	0.018	0.055	90.1	5.8

### Pesticide residues in fruits and vegetables

In this survey, 1146 samples of fruits and vegetables were analyzed for 400 pesticides contamination to assess health risk. In 1055 of 1146 analyzed fruit and vegetable samples (92.1%), no detectable residues were found, while pesticide residues were detected in 91 samples (8.9%). A number of 11 samples (1.0%) contained residues above MRLs established by the MFDS in Republic of Korea. Perilla leaves (13 samples, 11.4%), welsh onions (11 samples, 12.3%), chili peppers (7 samples, 16.7%), lettuce leaves (7 samples, 4.1%), aster scabers (6 samples, 19.6%), Chinese chives (6 samples, 12.2%) and winter-grown cabbages (6 samples, 3.8%) had a number of contaminated samples. Aster scabers (3 samples, 6.5%), pimpinella brachycarpas (3 samples, 8.3%), crown daisies (2 samples 2.6%), welsh onions (2 samples, 1.9%) and schisandraberries (1 sample, 16.7%) violated MRLs (Table 2). Szpyrka et al.^10^ reported that pesticide residues were detected in 36.6% of the analyzed in fruits and vegetables in Poland. In Republic of Korea, detectable residues were found in 13.9% of 34,520 samples of vegetables collected from 2010 to 2014^22^. In Algeria, pesticide residues monitoring for fruits and vegetables revealed residual pesticides in 57.5% of the analyzed samples^12^. Chen et al.^23^ found residues of selected fungicide and insecticides in vegetables including lettuce and spinach from Xiamen, China. Of the 147 samples of lettuce, pesticide residues were detected in the 52 samples (35.4%). In spinach, of the 55 samples, 18 samples (32.7%) were found to contain pesticide residues.

**Table 2:** Occurrence of pesticide residues in fruits and vegetables.

Group	Product	Sample	Without residue		With residue < MRL			With residue > MRL	
Vegetable	Aster scaber	46	37	80.4%	6		19.6%	3	6.5%
	Chard	29	28	96.6%	1		3.4%	0	0.0%
	Chili pepper	42	35	83.3%	7		16.7%	0	0.0%
	Chinese chives	49	43	87.8%	6		12.2%	0	0.0%
	Crown daisy	78	72	92.3%	4		7.7%	2	2.6%
	Giant butterbur	9	4	44.4%	5		55.6%	0	0.0%
	Lettuce leaves	171	164	95.9%	7		4.1%	0	0.0%
	Perilla leaves	114	101	88.6%	13		11.4%	0	0.0%
	Pimpinella brachycarpa	36	31	86.1%	2		13.9%	3	8.3%
	Spinach	132	130	98.5%	2		1.5%	0	0.0%
	Water-celery	79	75	94.9%	4		5.1%	0	0.0%
	Welsh onion	106	93	87.7%	11		12.3%	2	1.9%
	Winter-grown cabbage	158	152	96.2%	6		3.8%	0	0.0%
Fruit	Apple	22	19	86.4%	3		13.6%	0	0.0%
	Banana	18	16	88.9%	2		11.1%	0	0.0%
	Grape	20	19	95.0%	1		5.0%	0	0.0%
	Mandarin	9	9	100%	0		0.0%	0	0.0%
	Pear	14	14	100%	0		0.0%	0	0.0%
	Persimmon	8	8	100%	0		0.0%	0	0.0%
	Schisandraberry	6	5	83.3%	0		16.7%	1	16.7%
	Total	1,146	1,055	92.1%	80		7.9%	11	1.0%

### Incidences and MRL violation of pesticide residues

**Figure 1:**
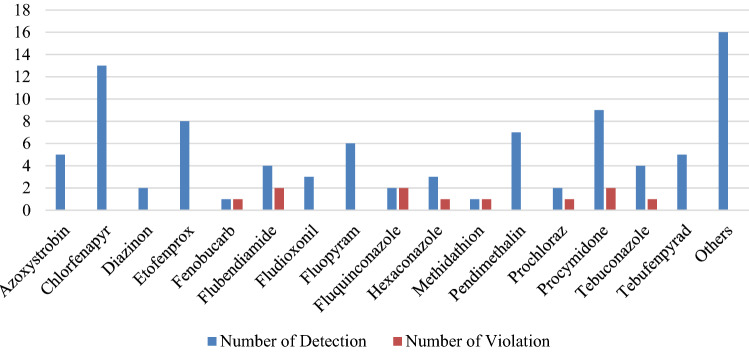
Number of pesticide residues detected in fruits and vegetables and number of excess MRLs.

Table 3 shows the frequency and concentration of pesticide residues in the analyzed samples. Of the 400 pesticides tested, 32 pesticides were detected in the analyzed samples. The numbers of pesticides detected by function was 15 fungicides (46.9%), 14 insecticides (43.7%), 2 herbicides (6.3%) and 1 growth regulator (3.1%). The most frequently detected pesticides in fruits and vegetables were chlorfenapyr (13 samples, 1.1%), procymidone (9 samples, 0.8%), etofenprox (8 samples, 0.7%), pendimethalin (7 samples, 0.6%) and fluopyram (6 samples, 0.5%). Theses pesticides accounted for approximately 47.3% of all pesticides detected in this study. Figure 1 shows the number of pesticide residues detected in fruits and vegetables and the number of excess MRLs. It was detected mainly in aster scabers, chili peppers, lettuce leaves, perilla leaves and welsh onions. The maximum residue concentration of fludioxonil was 7.48 mg/kg, which was the highest value among the detected pesticides. Chlorfenapyr was found in a concentration range of 0.116–1.452 mg/kg, procymidone in a range of 0.029–0.75 mg/kg, etofenprox in a range of 0.07–0.71 mg/kg, pendimethalin in a range of 0.024–0.069 mg/kg and fluopyram 0.010–0.130 mg/kg.

**Table 3:** Pesticide residues detected in fruits and vegetables.

Pesticide	Group	N > LOD (%)	N > MRL (%)	Range (mg/kg)	MRL (mg/kg)
Alachlor	H	1 (0.1%)	0 (0.0%)	0.038	0.05
Azoxystrobin	F	5 (0.4%)	0 (0.0%)	0.08–0.73	2.0–20
Chlorantraniliprole	I	1 (0.1%)	0 (0.0%)	0.283	2.0
Chlorfenapyr	I	13 (1.1%)	0 (0.0%)	0.116–1.452	1.0–5.0
Chlorfluazuron	I	1 (0.1%)	0 (0.0%)	0.32	5.0
Diazinon	I	2 (0.2%)	0 (0.0%)	0.020–0.023	0.05–0.1
Diethofencarb	F	1 (0.1%)	0 (0.0%)	0.93	5.0
Dimethoate	I	1 (0.1%)	0 (0.0%)	0.042	0.05
Etofenprox	I	8 (0.7%)	0 (0.0%)	0.07–0.71	1.0–7.0
Fenitrothion	I	1 (0.1%)	0 (0.0%)	0.015	0.05
Fenobucarb	I	1 (0.1%)	1 (0.1%)	0.364	0.01
Flubendiamide	I	4 (0.3%)	2 (0.2%)	0.058–2.8	0.02–15
Fludioxonil	F	3 (0.3%)	0 (0.0%)	0.13–7.48	5.0–40
Fluopyram	F	6 (0.5%)	0 (0.0%)	0.010–0.130	0.04–3.0
Fluquinconazole	F	2 (0.2%)	2 (0.2%)	0.260–0.712	0.05–0.3
Hexaconazole	F	3 (0.3%)	1 (0.1%)	0.58–1.82	0.7–1.0
Iprodione	F	1 (0.1%)	0 (0.0%)	2.3	20
Lufenuron	I	1 (0.1%)	0 (0.0%)	0.22	1.0
Methidathion	I	1 (0.1%)	1 (0.1%)	0.365	0.05
Myclobutanil	F	1 (0.1%)	0 (0.0%)	0.51	2.0
Paclobutrazol	G	1 (0.1%)	0 (0.0%)	0.18	0.5
Pendimethalin	H	7 (0.6%)	0 (0.0%)	0.024–0.069	0.05–0.07
Penthiopyrad	F	1 (0.1%)	0 (0.0%)	1.1	15
Prochloraz	F	2 (0.2%)	1 (0.1%)	0.07–1.86	1.0–5.0
Procymidone	F	9 (0.8%)	2 (0.2%)	0.029–0.75	0.05–5.0
Pyraclostrobin	F	1 (0.1%)	0 (0.0%)	0.17	1.0
Pyridalyl	I	1 (0.1%)	0 (0.0%)	0.08	5.0
Tebuconazole	F	4 (0.3%)	1 (0.1%)	0.041–0.390	0.05–3.0
Tebufenpyrad	I	5 (0.4%)	0 (0.0%)	0.10–0.72	5.0
Tebupirimfos	I	1 (0.1%)	0 (0.0%)	0.027	0.05
Tetraconazole	F	1 (0.1%)	0 (0.0%)	0.234	1.0
Thifluzamide	F	1 (0.1%)	0 (0.0%)	0.032	0.05

F: Fungicide, G: Growth regulator, H: Herbicide, I: Insecticide.

Of the 1,146 samples analyzed, 11 (1.0%) samples exceeded the MRLs. Fenobucarb, flubendiamide, fluquinconazole, hexaconazole, methidathion, prochloraz, procymidone and tebuconazole had residues that violated MRLs. Aster scabers, crown daisies, pimpinella brachycarpas, welsh onions and schisandraberries were the fruits and vegetables with residues above MRLs. Schisandraberries, pimpinella brachycarpas and aster scabers had high violations rate of 16.7%, 8.3% and 6.5%, respectively. The most frequently violated pesticides were flubendiamide, fluquinconazole and procymidone. The result of pesticide residues that violated the MRLs generally showed similar results to other studies in the Republic of Korea. In a report^24^ by the Gyeonggido Institute of Health and Environment, diazinon, carbofuran, fluquinconazole and procymidone were shown to have a high number of residues exceeding MRLs. And, Yi et al.^22^ reported that one of the pesticides frequently detected in excess of MRLs was diazinon, paclobutrazol and procymidone.

The percentage of samples with pesticide residues exceeding MRLs in this study (1.0%) was lower than the majority of those reported in other studies. Szpyrka et al.^10^ reported 1.8% exceedance of MRLs in fruits and vegetables collected in south-eastern Poland. According to a report by the United States Food and Drug Administration, pesticide residues exceeding the MRLs were detected in 2% of the domestic vegetables and 7% of the imported vegetable samples. Park et al.^25^ found that 1.4% of vegetables exceeded MRLs in Republic of Korea. A study from Algeria found that 12.5% of fruit and vegetable samples contained pesticide residues that exceeded the MRLs^12^. In addition, incidences of pesticide residues above MRL were reported to be 1.4% in vegetables collected from markets in Seoul, Republic of Korea^22^.

### Risk assessment

Pesticide residues in fruits and vegetables are unlikely to be completely removed by washing. Therefore, it is a very dangerous situation when consumers eat fruits and vegetables contaminated with high concentrations of pesticide residues over a long period time. The risk from pesticide residues in fruits and vegetables was performed on dietary exposure assessment for all detected pesticide in the samples. The results of human exposure to pesticides based on fruit and vegetable intake are shown in Table 4. In the short term risk assessment, the ESTIs of pesticide range from 1.2 × 10^–7^ to 1.4 × 10^–2^ mg/kg bw/day. The range of aHQ was 0.000–8.411%. The highest values of aHQ were obtained in case of consumption of lettuce (leaves) with chlorfenapyr. In a similar study, Mebdoua et al.^12^ identified a potential acute exposure for pesticide residues in fruits and vegetables for the population of Algerian. The values of short term exposure ranged from 0.78% to 558.5% of aHQ for children and ranged from 0.23% to 237.8% of aHQ for adults. In the long term risk assessment, the EDIs of pesticide range from 3.7 × 10^–9^ to 7.0 × 10^–4^ mg/kg bw/day. The range of HQs was 0.000–6.384%. The HQs for fluquinconazole and prochloraz were 0.080–6.384 and 0.131–3.336%, respectively and were higher than those of other pesticides. The HQ value above 100% indicates a potential risk to consumers^20,26^. Therefore, the results indicate that the detected pesticides in this study are not harmful to human health. The cHI for all residues was 17.714%, which was less than 100%, meaning that there is no risk of side effects following a cumulative exposure to all the detected pesticides. Elgueta et al.^27^ reported chronic health risk for consumers in leafy vegetables. They found HQs values for methamidophos (73.9%), cypermethrin (30.4%), mancozeb (11.5%) and cyfluthrin (4.5%), etc. The HQs were summed up and the cHI of all residues was 135%, more than 100%. Chen et al.^23^ detected pesticide residues in fruits and vegetables, but there was no health risk for consumers. They found HQ values for omethoate (2.6%), methamidophos (2.2%) and chlorpyrifos (0.24%). Since pesticide residues are reduced by 8–68% with flowing water at home depending on the type of pesticide residues and characteristics of fruits and vegetables, the risk of pesticide residues can be lowered^28^. Additionally, agricultural products in excess of MRLs are seized by the government and unsuitable agricultural products are immediately discarded in Republic of Korea. However, continuous management and monitoring of agricultural products is required for the safety of consumers because intake of agricultural products changes according to consumer’s preference, region and season.

**Table 4:** Risk assessment for detected pesticides in fruits and vegetables.

Pesticide	Fruit and vegetable	Short term risk assessment			Long term risk assessment		
		ARfD (mg/kg bw)	ESTI (mg/kg bw/day)	aHQ (%)	ADI (mg/kg bw/day)	EDI (mg/kg bw/day)	HQ (%)
Alachor	Welsh onion	–	2.5 × 10^–5^	–	0.01	6.8 × 10^–6^	0.068
Azoxystrobin	Banana	–	2.9 × 10^–4^	–	0.2	1.5 × 10^–5^	0.007
	Grape	–	6.3 × 10^–4^	–	0.2	2.0 × 10^–5^	0.010
	Spinach	–	1.3 × 10^–3^	–	0.2	6.4 × 10^–5^	0.032
	Welsh onion	–	4.2 × 10^–4^	–	0.2	6.8 × 10^–5^	0.034
Chlorantraniliprole	Welsh onion	–	1.8 × 10^–4^	–	2	5.1 × 10^–5^	0.003
Chlorfenapyr	Aster scaber	0.03	5.1 × 10^–4^	1.701	0.026	8.3 × 10^–6^	0.032
	Chili pepper	0.03	1.1 × 10^–4^	0.372	0.026	1.0 × 10^–5^	0.039
	Chinese chives	0.03	2.6 × 10^–4^	0.865	0.026	9.8 × 10^–6^	0.038
	Crown daisy	0.03	3.1 × 10^–5^	0.103	0.026	1.5 × 10^–6^	0.006
	Giant butterbur	0.03	2.4 × 10^–4^	0.813	0.026	6.6 × 10^–6^	0.025
	Lettuce(leaves)	0.03	2.5 × 10^–3^	8.411	0.026	8.2 × 10^–5^	0.314
Chlorfluazuron	Chard	–	4.1 × 10^–6^	–	0.033	1.0 × 10^–6^	0.003
Diazinon	W. cabbage	0.025	1.2 × 10^–7^	0.000	0.0002	3.7 × 10^–9^	0.002
Diethofencarb	P. brachycarpa	–	8.4 × 10^–4^	–	0.43	5.7 × 10^–6^	0.001
Dimethoate	Welsh onion	0.02	2.7 × 10^–5^	0.137	0.002	7.5 × 10^–6^	0.377
Etofenprox	Apple	1	2.8 × 10^–3^	0.276	0.03	1.2 × 10^–4^	0.410
	Chinese chives	1	7.7 × 10^–4^	0.077	0.03	3.8 × 10^–5^	0.126
	Water-cerery	1	3.2 × 10^–4^	0.032	0.03	6.0 × 10^–6^	0.020
	Welsh onion	1	1.4 × 10^–4^	0.014	0.03	3.8 × 10^–5^	0.126
	W. cabbage	1	1.4 × 10^–6^	0.000	0.03	4.7 × 10^–8^	0.000
Fenitrothion	Crown daisy	0.04	2.7 × 10^–6^	0.007	0.005	1.3 × 10^–7^	0.003
Fenobucarb	Aster scaber	–	1.9 × 10^–4^	–	0.014	7.6 × 10^–6^	0.055
Flubendiamide	Crown daisy	0.2	8.0 × 10^–5^	0.040	0.017	1.3 × 10^–7^	0.001
	Perilla leaves	0.2	8.5 × 10^–4^	0.427	0.017	1.1 × 10^–4^	0.622
Fludioxonil	Perilla leaves	–	2.3 × 10^–3^	–	0.4	2.2 × 10^–4^	0.056
	Water-cerery	–	6.8 × 10^–5^	–	0.4	2.4 × 10^–6^	0.001
Fluopyram	Chili pepper	0.5	1.0 × 10^–4^	0.020	0.01	9.2 × 10^–6^	0.092
	Crown daisy	0.5	4.0 × 10^–6^	0.001	0.01	1.9 × 10^–7^	0.002
	Lettuce(leaves)	0.5	2.1 × 10^–4^	0.042	0.01	8.7 × 10^–6^	0.087
	Welsh onion	0.5	6.5 × 10^–6^	0.001	0.01	1.8 × 10^–6^	0.018
Fluquinconazole	P. brachycarpa	0.02	2.3 × 10^–4^	1.171	0.002	1.6 × 10^–6^	0.080
	Welsh onion	0.02	4.6 × 10^–4^	2.314	0.002	1.3 × 10^–4^	6.384
Hexaconazole	Aster scaber	0.25	9.3 × 10^–4^	0.370	0.005	2.7 × 10^–5^	0.533
	W. cabbage	0.25	2.9 × 10^–6^	0.001	0.005	9.7 × 10^–8^	0.002
Iprodione	Perilla leaves	0.06	7.0 × 10^–4^	1.169	0.06	1.1 × 10^–4^	0.176
Lufenuron	W. cabbage	–	1.1 × 10^–6^	–	0.015	3.7 × 10^–8^	0.000
Methidathion	Schisandraberry	0.01	4.3 × 10^–5^	0.431	0.001	1.8 × 10^–7^	0.018
Myclobutanil	Aster scaber	–	2.6 × 10^–4^	–	0.03	1.1 × 10^–5^	0.0036
Paclobutrazol	Chinese chives	0.1	1.9 × 10^–4^	0.195	0.022	9.6 × 10^–6^	0.044
Pendimethalin	Giant butterbur	–	4.5 × 10^–6^	–	0.13	1.5 × 10^–7^	0.000
	Perilla leaves	–	7.6 × 10^–6^	–	0.13	1.2 × 10^–6^	0.001
	Welsh onion	–	2.7 × 10^–5^	–	0.13	6.3 × 10^–6^	0.005
	W. cabbage	–	3.5 × 10^–7^	–	0.13	1.2 × 10^–8^	0.000
Penthiopyrad	Perilla leaves	–	3.4 × 10^–4^	–	0.018	5.1 × 10^–5^	0.281
Prochloraz	Banana	0.1	2.6 × 10^–4^	0.285	0.01	1.3 × 10^–5^	0.131
	Welsh onion	0.1	1.2 × 10^–3^	1.209	0.01	3.3 × 10^–4^	3.336
Procymidone	Chili pepper	0.1	2.2 × 10^–4^	0.220	0.1	1.7 × 10^–5^	0.017
	Chinese chives	0.1	8.1 × 10^–4^	0.811	0.1	3.8 × 10^–5^	0.038
	Crown daisy	0.1	5.3 × 10^–6^	0.005	0.1	2.5 × 10^–7^	0.000
	P. brachycarpa	0.1	1.1 × 10^–4^	0.110	0.1	5.8 × 10^–7^	0.001
	Welsh onion	0.1	1.2 × 10^–4^	0.117	0.1	3.2 × 10^–5^	0.032
Pyraclostrobin	Chili pepper	0.05	1.3 × 10^–4^	0.267	0.03	1.2 × 10^–5^	0.040
Pyridalyl	Spinach	–	1.4 × 10^–2^	–	0.028	7.0 × 10^–4^	2.495
Tebuconazole	Apple	0.3	5.5 × 10^–4^	0.1843	0.03	4.0 × 10^–5^	0.133
	Aster scaber	0.3	2.0 × 10^–4^	0.0661	0.03	8.2 × 10^–6^	0.027
	Chili pepper	0.3	2.3 × 10^–4^	0.0760	0.03	2.0 × 10^–5^	0.068
	Lettuce(leaves)	0.3	7.1 × 10^–5^	0.0238	0.03	4.2 × 10^–6^	0.014
Tebufenpyrad	Perilla leaves	0.02	2.2 × 10^–4^	1.0980	0.01	1.6 × 10^–5^	0.162
Tebupirimfos	Perilla leaves	–	8.1 × 10^–6^	–	0.0002	1.2 × 10^–6^	0.614
Tetraconazole	Chili pepper	0.05	1.8 × 10^–4^	0.3681	0.004	1.7 × 10^–5^	0.413
Thifluzamide	Lettuce(leaves)	–	5.6 × 10^–5^	–	0.014	3.3 × 10^–6^	0.023

The “–” symbol indicates that there was no authorized ARfD value and the corresponding risk index could not be calculated.

In conclusion, pesticide residues were found in 8.9% of the samples and exceeded the MRLs in 1.0% of the total fruits and vegetables. The frequently detected pesticides were chlorfenapyr, procymidone, etofenprox, pendimethalin and fluopyram, while the high rate of violations were flubendiamide, fluquinconazole and procymidone. Based on the findings, the range of aHQs and HQs was 0.000–8.411 and 0.000–6.384%, respectively. Therefore, the results showed that the consumers were not exposed to health risks through the consumption of fruits and vegetables. The results provide important information about the current state of pollution in fruits and vegetables. The obtained data can be used to develop strategies and improve pesticide MRLs for the safe management of fruits and vegetables in Republic of Korea.

## Supplementary Information

Supplementary Information.

## Data Availability

All data generated and/ or analyzed during this study are available from the corresponding author on reasonable request.
