# Supplementary material for: Detection of pesticide residues and risk assessment from the local fruits and vegetables in Incheon, Korea
Source: Sci Rep. 2022 Jun 10;12:9613. doi: 10.1038/s41598-022-13576-5 (PMC9187649; doi:10.1038/s41598-022-13576-5)
Supplement: Supplementary file 1 — Supplementary Information. [file 41598_2022_13576_MOESM1_ESM.pdf]

< Supplementary Information >

Title

Detection of pesticide residues and risk assessment from the local fruits and vegetables in Incheon, Korea

Author list

Byung Kyu Park

Sung Hee Kwon

Mi Sook Yeom

Kwang Sig Joo

Myung Je Heo

Table S1. List of pesticides analyzed in this study

Instrument	Pesticide and metabolite
GC-MS/MS	2,6-Diisoporpylnaphthalene, Alachlor, Allethrin, Allidochlor, Aspon, Azinphos-ethyl, Benalaxyl, Benodanil, Benzoylprop-ethyl, Bifenox, Bromophos-methyl, Butachlor, Butralin, Butylate, Chlorbufam, Chlordane-trans, Chlorethoxyfos, Chlorfenson, Chlorfluazuron, Chlorflurenol-methyl, Chloroneb, Chloropropylate, Chlorpropham, Chlorthion, Chlorthiophos, Chlozolinate, Cinmethylin, Clomazone, Cycloate, Cyhalofop-butyl, Demeton-O, Demeton-S, Demeton-S-methyl-sulfone, Desmetryn, Diallate, Dichlofenthion, Dichlormid, Diclofop-methyl, Diethatyl-ethyl, Dimethametryn, Dinitramine, Dioxathion, Epoxiconazole, EPTC, Etaconazole, Ethalfluralin, Ethofumestae, Ethychlozate, Etofenprox, Etridiazole, Fenchlorphos, Fenclorim, Fenfuram, Fenson, Flamprop-isopropyl, Fluazifop-butyl, Fluchloralin, Flufenpyr-ethyl, Flumetralin, Flumioxazine, Fluopyram, Fluorodifen, Flurochloridone, Flutamone, Flutianil, Flutriafol, Formothion, Halfenprox, Heptenophos, Hexaconazole, Isazofos, Isopropalin, Isotianil, Leptophos, Methoprotryne, Methyl trithion, Metolachlor, Metribuzin, MGK, Napropamide, Norflurazon, Oxydemeton-methyl, Oxyfluorfen, Pebulate, Penflufen, Penthiopyrad, Pentoxazone, Perthane, Pretilachlor, Profluralin, Prometon, Prometryn, Propachlor, Propanil, Propetamphos, Propham, Pyracarbolid, Pyrifenox, Quinoxyfen, Secbumeton, Silafluofen, Simetryn, Spiromesifen, Spiroxamine, Sulfotep, Sulprofos, Terbacil, Terbumeton, Terbutryn, Tetrachlorvinphos, Tetraconazole, Tetramethrin, Tetrasul, Tridadimenol, Triallate, Tribufos, Trifluralin,
GC-ECD	Acrinathrin, Aldrin, Anilofos, BHC, Bifenthrin, Binapacryl, Bromacil, Bromopropylate, Butafenacil, Captafol, Captan, Chlordane, Chloridazon, Chlorobenzilate, Chlorothalonil, Chlortal-dimethyl, Clomeprop, Cyanazine, Cyflufenamid, Cyfluthrin, Cyhalothrin, Cypermethrin, DDT, Deltamethrin, Dicloran, Dicofol, Dieldrin, Diflufenican, Dimepiperate, Dimethachlor, Diniconazole, Dithiopyr, Edifenphos, Endosulfan, Endrin, Fenthion, Fenvalerate, Flonicamid, Flumiclorac-pentyl, Fluthiacet-methyl, Fluvalinate, Folpet, Fthalide, Heptachlor, Heptachlorepoxide, Hexachlorbenzene, Indanofan, Indoxacarb, Iprodione, Iprovalicarb, Isoprothiolane, Lactofen, Lindane, Mefenacet, Mefenpyr-diethyl, Methoxychlor, Metrafenone, Nitrapyrin, Nitrothal-isopropyl, cis-Nonachlor, trans-Nonachlor, Nuarimol, Ofurace, Permethrin, Picolinafen, Picoxystrobin, Probenazole, Prochloraz, Pronamide, Pyrimidifen, Quintozene, TCMTB, Tefluthrin, Tetradifon, Triflumuron, Uniconazole, Vernolate
GC-NPD	Ametryn, Atrazine, Azaconazole, Bromobutide, Bupirimate, Cadusafos, Carbophenothion, Chinomethionat, Chlorfenapyr, Chlorpyrifos, Chlorpyrifos-methyl, Cyanophos, Cyproconazole, Diazinon, Dichlofluanid, Dichlorvos, Diethofencarb, Dimetenamid, Dimethoate, Dimethylvinphos, Diphenmaid, Diphenylamine, EPN, Esprocarb, Ethion, Ethoprophos, Etoxazole, Etrimfos, Fenamidone, Fenarimol, Fenbuconazole, Fenitrothion, Fenobucarb, Fenoxanil, Fenoxycarb, Fenpropathrin, Fipronil, Fludioxonil, Flusilazole, Flutolanil, Fonofos, Fosthiazate, Furathiocarb, Iprobenfos, Isofenphos, Isofenphos-methyl, Isoxanthion, Kresoxim-methyl, Malathion, Mecarbam, Mepronil, Metconazole, Methidathion, Molinate, Myclobutanil, Oxadixyl, Paclobutrazol, Parathion-ethyl, Parathion-methyl, Penconazole, Pendimethalin, Phenthoate, Phosalone, Phosmet, Phosphamidone, Piperophos, Pirimicarb, Pirimiphos-ethyl, Pirimiphos-methyl, Procymidone, Profenofos, Propazine, Propisochlor, Prothiofos, Pyraclofos, Pyrazophos, Pyridaben, Pyridalyl, Pyriminobac-methyl, Quinalphos, Simeconazole, Tebuconazole, Tebufenpyrad, Tebupirimfos, Terbufos, Terbuthylazine, Thiazopyr, Thifluzamide, Thiometon, Tolfenpyrad, Tolylfluanid, Tralomethrin, Triadimefon, Triazophos, Triflumizole, Vinclozolin, Zoxamide
LC-MS/MS	Aldicarb, Azamethiphos, Bendiocarb, Bensulide, Bixafen, Butocarboxim, Carbaryl, Carbetamide, Crufomate, Cycloprothrin, Dicrotophos, Ethiofencarb, Fluazinam, Fluometuron, Fluridone, Hexazinone, Hexythiazox, Imidacloprid, Ipconazole, Isoprocarb, Isoxaben, Lenacil, Malaoxon, Mephosfolan, Metamitron, Methiocarb, Methomyl, Metolcarb, Metominostrobin, Neburon, Noruron, Pinoxaden, Promecarb, Propaquizafop, Propoxur, Prosulfocarb, Prothioconazole, Pyraclonil, Pyraflufen-ethyl, Simazine, Sulfentrazone_NH4, Tebuthiuron, Tepraloxydim, Thiodicarb, Tridemorph, 2,3,5-Trimethacarb, Triticonazole, XMC
LC-UVD	Amisulbrom, Azoxystrobin, Benzoximate, Boscalid, Bromoxynil, Chlorantraniliprole, Chlorimuron-ethyl, Chlorobenzuron, Chlorotoluron, Chromafenozide, Cinosulfuron, Cyazofamid, Cymoxanil, Dimethomorph, Ethaboxam, Ethametsulfuron-methyl, Fenhexamid, Fenpyroximate, Ferimzone, Fluacrypyrim, Flubendiamide, Flufenacet, Flufenoxuron, Fluquinconazole, Flusulfamide, Forchlorfenuron, Hexaflumuron, Imazamox, Imazapic, Imazaquin, Imazethapyr, Imibenconazole, Isoproturon, Lufenuron, Mepanipyrim, Metamifop, Methabenzthiazuron, Methoxyfenozide, Nitenpyram, Novaluron, Oxaziclomefone, Phenmedipham, Pyraclostrobin, Pyrazolate, Pyribenzoxim, Pyributicarb, Pyridate, Pyrimethanil, Pyriproxyfen, Pyroquilon, Quinoclamine, Rimsulfuron, Spriodiclofen, Tebufenozide, Teflubenzuron, Thenylchlor, Thiamethoxam, Tiadinil, Tribenuron-methyl, Tricyclazole, Trifloxystrobin,

Table S2. Identification criteria ion for GC-MS/MS analysis

Pesticide	Precursor ion (*m/z*)	Production ion (*m/z*)	RT (min)	Collision energy (eV)
2,6-Diisoporpylnaphthalene	212	197	13.03	15
	197	167	13.03	10
Acrinathrin	289	93	26.12	20
	289	91	26.12	20
Alachlor	188	160	15.17	10
	188	131	15.17	20
Aldrin	263	228	16.01	20
	263	193	16.01	40
Allethrin	123	95	17.72	10
	123	81	17.72	10
	123	79	17.72	10
Allidochlor	138	96	8.79	10
	132	56	8.79	5
Ametryn	227	185	15.34	5
	227	170	15.34	10
Anilofos	226	184	24.38	10
	226	157	24.38	20
Aspon	210.8	115	16.12	10
	209.8	145.9	16.12	10
Atrazine	215	200	13.18	5
	215	58	13.18	10
Azaconazole	217	217	19.84	5
	217	173	19.84	20
Azinphos-ethyl	160	77	26.28	20
	132	77	26.28	15
Benalaxyl	148	105.1	21.68	20
	148	77	21.68	35
Benodanil	230.9	203	21.20	15
	230.9	76.1	21.20	25
Benzoylprop-ethyl	105	77.1	23.26	15
	105	51.1	23.26	35
BHC-alpha	219	183	12.72	10
	219	145	12.72	20
BHC-beta	219	183	13.26	10

	219	145	13.26	20
BHC-delta	219	183	13.50	10
	219	145	13.50	20
Bifenox	341	310	24.43	10
	341	281	24.43	15
Bifenthrin	181	166	23.87	10
	181	165	23.87	30
Binapacryl	83	83	19.98	5
	83	55	19.98	5
Bromacil	205	188	15.88	15
	205	54	15.88	40
Bromobutide	119	91	14.96	10
	119	65	14.96	30
Bromophos-methyl	330.8	315.8	16.96	15
	125	47	16.96	10
Bromopropylate	341	185	23.90	20
	341	155	23.90	40
Bupirimate	273	193	19.69	10
	273	109	19.69	15
Butachlor	188	160	18.59	8
	176	147	18.59	12
Butafenacil	331	331	27.89	5
	331	180	27.89	10
Butralin	266	220.2	16.81	10
	266	174.2	16.81	20
Butylate	156	57.1	9.92	5
	146.1	90	9.92	5
Cadusafos	159	131	12.45	5
	159	97	12.45	20
Captafol	151	122	22.90	10
	151	80	22.90	10
	151	79	22.90	20
	151	77	22.90	40
	79	77	22.90	15
	79	51	22.90	25
Captan	149	148	17.92	10
	149	107	17.92	10
	149	105	17.92	10

	149	79	17.92	20
	149	77	17.92	40
	149	70	17.92	20
	79	78	17.92	10
	79	77	17.92	20
Carbophenothion	342	157	21.79	15
	157	45	21.79	15
Chinomethionat	206	148	18.41	20
	206	121	18.41	30
Chlorbufam	153	125.1	13.17	10
	153	90	13.17	20
Chlordane-cis	373	266	18.37	20
	272	237	18.37	15
Chlordane-trans	373	266	18.78	20
	272	237	18.78	15
Chlorethoxyfos	153	97	11.68	10
	97	65	11.68	15
Chlorfenapyr	328	247	19.91	15
	247	227	19.91	20
	247	200	19.91	30
	247	197	19.91	20
Chlorfenson	175	111	19.13	10
	111	75	19.13	15
Chlorfluazuron	321	304	18.82	10
	321	286	18.82	10
	321	113	18.82	40
	321	97	18.82	30
Chlorflurenol-methyl	217	152	18.16	25
	215	152	18.16	20
Chloridazon	221	220	22.07	10
	221	105	22.07	15
Chlorobenzilate	251	111	20.58	40
	251	75	20.58	45
Chloroneb	206	191.1	10.59	10
	191	113	10.59	15
Chloropropylate	251.1	139.1	20.58	15
	139.1	111	20.58	15
Chlorothalonil	266	170	15.45	30

	266	133	15.45	40
Chlorpropham	213	171	12.12	10
	171	127	12.12	15
Chlorpyrifos	197	169	16.28	15
	197	107	16.28	40
Chlorpyrifos-methyl	286	271	14.94	15
	286	93	14.94	25
Chlorthal-dimethyl	301	273	16.43	15
	301	222	16.43	25
Chlorthion	297	109	16.75	15
	297	79	16.75	40
Chlorthiophos	324.8	268.9	20.95	10
	296.8	268.9	20.95	5
Chlozolinate	188.1	147	17.52	15
	186	145	17.52	15
Cinmethylin	123	123	15.44	5
	123	81	15.44	10
Clomazone	204	107	13.33	25
	125	89	13.33	15
Clomeprop	288	132	24.59	25
	288	120	24.59	30
Cyanazine	212	151	16.39	15
	212	123	16.39	20
Cyanophos	243	148	13.55	5
	243	109	13.55	15
Cycloate	154	83	11.94	10
	154	55	11.94	20
Cyflufenamid	294	237	20.05	10
	294	225	20.05	10
	294	223	20.05	10
	294	203	20.05	30
	91	65	20.05	20
Cyfluthrin	226	206	28.51	10
	226	199	28.51	15
Cyhalofop-butyl	256.2	120	25.48	10
	229.2	109	25.48	15
Cyhalothrin	197	161	25.74	5
	197	141	25.74	10

Cyhalothrin-1	197	161	25.41	5
	197	141	25.41	10
Cypermethrin	163	127	29.14	6
	163	91	29.14	15
Cyproconazole	222	125	20.20	20
	222	82	20.20	10
p,p’-DDD	237	165	16.40	25
	235	165	16.40	25
p,p’-DDE	246	176	15.06	30
	248	176	15.06	30
o,p’-DDT	237	165	16.47	25
	235	165	16.47	25
p,p’-DDT	237	165	17.64	25
	235	165	17.64	25
Deltamethrin	253	93	32.32	20
	253	77	32.32	40
	181	152	32.32	30
	181	127	32.32	30
Demeton-O	171	115	11.60	10
	88	60	11.60	5
Demeton-S	170	114	12.92	10
	88	60	12.92	5
Demeton-S-methyl-sulfone	169	154	14.00	10
	169	112	14.00	20
	169	111	14.00	20
	169	69	14.00	40
Desmetryn	213	171.2	14.76	5
	213	58.1	14.76	10
Diallate	234	192	12.53	10
	234	150	12.53	25
Diazinon	179	137	13.64	20
	179	121	13.64	40
Dichlofenthion	279	223	14.77	15
	223	205	14.77	15
Dichlofluanid	224	123	16.05	20
	123	77	16.05	20
	123	51	16.05	40
Dichlormid	172.1	108.1	9.33	5

	166	56.1	9.33	10
Dichlorvos(DDVP)	109	109	8.43	5
	109	79	8.43	5
Diclofop-methyl	340	253	22.68	10
	281	120	22.68	15
Dicloran	206	176	12.98	10
	206	124	12.98	30
Dicofol	139	111	16.78	10
	139	75	16.78	30
Dieldrin	263	193	19.67	40
	79	77	19.67	20
	79	51	19.67	30
Diethatyl-ethyl	188.1	160.2	18.80	5
	162.1	147.1	18.80	10
Diethofencarb	207	151	16.31	15
	151	123	16.31	10
Diflufenican	266	246	22.74	15
	266	238	22.74	15
Dimepiperate	119	91	17.98	10
	119	65	17.98	30
Dimethachlor	197	148	14.79	10
	197	120	14.79	20
Dimethametryn	212	142	17.50	10
	212	122	17.50	5
Dimethenamid	154	137	14.80	10
	154	111	14.80	10
Dimethoate	125	79	12.85	10
	125	47	12.85	20
Dimethylvinphos	297	109	16.34	15
	295	109	16.34	15
Diniconazole	268	232	20.73	10
	268	136	20.73	45
Dinitramine	260.7	241	13.87	10
	260.7	195	13.87	20
Dioxathion	271	197	13.43	10
	271	169	13.43	10
	271	141	13.43	20
	271	97	13.43	40

Diphenamid	167	165	16.98	20
	167	152	16.98	20
Diphenylamine	169	168	11.84	10
	169	167	11.84	30
Dithiopyr	354	306	15.45	5
	354	286	15.45	20
Edifenphos	173	109	21.88	5
	173	65	21.88	40
Endosulfan-alpha	195	159	18.79	10
	195	125	18.79	20
Endosulfan-beta	195	159	20.72	10
	195	125	20.72	30
Endosulfan-sulfate	272	237	21.99	10
	272	117	21.99	40
Endrin	263	228	20.36	20
	263	193	20.36	40
	263	191	20.36	30
EPN	157	110	23.84	20
	157	77	23.84	20
Epoxiconazole	192	138.1	23.10	10
	192	111	23.10	25
EPTC	132	90	9.34	5
	128	86	9.34	5
Esprocarb	222	162	16.09	5
	222	91	16.09	20
Etaconazole	173	145	19.84	15
	173	109	19.84	30
Ethalfluralin	315.9	275.9	12.07	10
	275.9	202.1	12.07	15
Ethion	231	175	20.89	10
	231	129	20.89	20
Ethofumesate	206.9	161.1	15.87	5
	161	105.1	15.87	10
Ethoprophos	158	114	12.45	5
	158	97	12.45	20
Ethychlozate	165	138	17.50	10
	165	102	17.50	15
Etofenprox (Ethofenprox)	163	135.1	29.56	10

	163	107.1	29.56	25
Etoxazole	141	113	24.14	15
	141	63	24.14	30
Etridiazole	210.9	182.9	10.14	5
	182.9	139.9	10.14	15
Etrimfos	292	181	14.07	10
	292	153	14.07	20
Fenamidone	238	103	24.21	20
	238	91	24.21	30
Fenarimol	139	111	26.01	15
	139	75	26.01	30
Fenbuconazole	129	102	28.18	20
	129	78	28.18	20
Fenchlorphos(Ronnel)	285	270	15.42	15
	285	93	15.42	20
Fenclorim	224	189	12.74	20
	189	104	12.74	10
Fenfuram	201	109	14.05	10
	109	53	14.05	15
Fenitrothion	277	260	15.82	5
	277	109	15.82	20
Fenobucarb	121	103	11.55	15
	121	91	11.55	15
Fenoxanil (deger)	189	154	20.24	10
	189	125	20.24	15
Fenoxycarb	255	186	24.02	10
	186	109	24.02	15
Fenpropathrin	265	210	24.20	20
	265	89	24.20	40
Fenson	268	77	16.95	20
	141	77	16.95	5
Fenthion	278	169	16.42	20
	278	109	16.42	20
Fenvalerate	225	147	31.04	10
	225	119	31.04	10
	167	125	31.04	10
	167	89	31.04	40
Fipronil	367	255	17.38	25

	367	213	17.38	25
Flamprop-isopropyl	105	77.1	24.35	15
	105	51.1	24.35	30
Flonicamid	174	146	11.35	15
	174	126	11.35	25
Fluazifop-butyl	282	238	20.35	25
	282	91	20.35	5
Fluchloralin	325.8	62.9	13.65	15
	306	263.9	13.65	10
Fludioxonil	248	182	19.13	20
	248	154	19.13	20
Flufenpyr-ethyl	408	373	20.53	10
	408	345	20.53	10
Flumetralin	143	117	18.50	20
	143	107.1	18.50	20
Flumiclorac-pentyl	318	260	32.58	15
	308	280	32.58	10
	308	79	32.58	30
Flumioxazine	354	325.9	30.54	5
	287	258.7	30.54	15
Fluopyram	223	187	17.64	20
	173	145	17.64	20
Fluorodifen	190	126.1	19.00	10
	190	75	19.00	20
Flurochloridone	311	174.1	16.75	15
	187.1	159.1	16.75	10
Flurtamone	332.7	120	24.75	15
	199	157.1	24.75	20
Flusilazole	233	165	19.67	25
	233	152	19.67	15
Fluthiacet-methyl	403	84	34.77	15
	403	83	34.77	15
Flutianil	231	216	29.77	5
	231	200	29.77	15
Flutolanil	173	173	19.06	5
	173	145	19.06	20
Flutriafol	123	95	18.91	15
	123	75	18.91	25

Fluvalinate	250	200	31.03	25
	250	55	31.03	20
Folpet	260	130	18.09	15
	260	102	18.09	45
Fonofos	246	137	13.68	5
	246	109	13.68	15
Formothion	126	93	14.52	5
	125	79	14.52	10
Fosthiazate	195	103	17.10	10
	195	60	17.10	20
Fthalide	243	215	16.84	20
	243	179	16.84	30
Furathiocarb	163	135	24.71	5
	163	107	24.71	15
Halfenprox	265	115.1	29.06	20
	262.9	115	29.06	20
Heptachlor	272	237	15.43	15
	272	117	15.43	45
Heptachlor epoxide	353	282	17.63	15
	353	263	17.63	15
Hepthenophos	124	89	11.19	20
	124	63	11.19	30
Hexachlorobenzene	284	249	12.81	25
	284	213	12.81	25
Hexaconazole	214	172	19.17	20
	214	159	19.17	20
Indanofan	174	159	24.35	10
	174	77	24.35	40
Indoxacarb	203	106	32.09	25
	203	78	32.09	40
	150	123	32.09	20
	150	88	32.09	40
Iprobenfos	204	171	14.37	5
	91	65	14.37	20
Iprodione	314	245	23.51	10
	314	56	23.51	20
Iprovalicarb	134	93	19.89	20
	134	42	19.89	20

	119	91	19.89	15
	116	98	19.89	5
Isazofos	161	146	13.98	10
	161	119	13.98	10
Isofenphos	213	185	17.57	5
	213	121	17.57	20
Isofenphos-methyl	199	121	17.13	15
	199	93	17.13	25
Isopropalin	264	222.2	17.08	5
	238	165.2	17.08	10
Isoprothiolane	162	134	19.25	5
	162	85	19.25	20
Isotianil	297	180	21.98	20
	180	91	21.98	15
Isoxathion	177	130	20.12	5
	177	116	20.12	10
Kresoxim-methyl	116	89	19.74	20
	116	63	19.74	30
Lactofen	344	223	25.77	20
	344	167	25.77	30
Leptophos	171	77	25.03	15
	155	77	25.03	15
Lindane	219	183	14.18	10
	219	145	14.18	20
Malathion	173	117	16.07	10
	173	99	16.07	10
Mecarbam	131	86	17.69	10
	131	74	17.69	10
Mefenacet	192	136	25.45	20
	192	109	25.45	30
Mefenpyr-diethyl	253	189	23.19	30
	253	163	23.19	30
Mepronil	269	119	21.34	10
	269	91	21.34	40
Metconazole	125	99	24.48	20
	125	89	24.48	20
Methidathion	145	85	18.27	5
	145	58	18.27	20

Methoprotryne	256	212	19.86	15
	256	170	19.86	25
Methoxychlor	227	169	24.08	30
	227	141	24.08	40
Methyl trithion	157	121	20.56	35
	157	75	20.56	40
Metolachlor	238	162	16.23	10
	162	133	16.23	15
Metrafenone	395	365	26.43	15
	393	363	26.43	10
Metribuzin	198	89	14.95	15
	198	55	14.95	30
MGK-264	164	98	17.42	10
	164	80	17.42	30
Molinate	126	83	11.01	5
	126	55	11.01	10
Myclobutanil	179	125	19.60	20
	179	90	19.60	40
Napropamide	271	72	19.01	15
	128	72	19.01	5
Nitrapyrin	194	158	10.13	20
	194	133	10.13	20
Nitrothal-isopropyl	236	194	16.73	10
	236	148	16.73	20
Nonachlor cis	407	300	20.85	20
	407	144	20.85	20
Nonachlor-trans	409	301	18.86	25
	409	263	18.86	25
Norflurazon	303	145	21.77	10
	145	75	21.77	30
Nuarimol	235	139	22.54	20
	235	123	22.54	20
Ofurace	232	186	21.44	10
	232	158	21.44	20
Oxadixyl	163	132	20.80	5
	163	117	20.80	25
Oxydemeton-methyl	109	109	7.52	5
	109	79	7.52	10

Oxyfluorfen	361	300	19.67	20
	300	223	19.67	20
Paclobutrazol	236	167	18.54	10
	236	125	18.54	10
Parathion-ethyl	291	109	16.53	8
	291	81	16.53	22
Parathion-methyl	263	109	15.13	10
	263	79	15.13	30
Pebulate	128	72	10.19	0
	128	57	10.19	5
Penconazole	248	192	17.50	20
	248	157	17.50	30
Pendimethalin	252	191	17.29	5
	252	162	17.29	10
Penflufen	274	141	21.24	15
	141	84	21.24	15
Penthiopyrad	302	177	20.77	20
	177	101	20.77	20
Pentoxazone	286.8	70	24.99	10
	284.8	70	24.99	10
Permethrin	183	168	27.25	15
	183	153	27.25	20
Permethrin-1	183	168	27.51	15
	183	153	27.51	20
Perthane	223	193	20.35	20
	223	165	20.35	20
Phenthoate	274	121	17.80	10
	274	93	17.80	10
Phosalone	182	111	25.00	10
	182	75	25.00	40
Phosmet	160	133	23.72	10
	160	77	23.72	25
Phosphamidone	264	127	14.69	15
	127	109	14.69	10
	127	95	14.69	15
Picolinafen	376	266	23.94	10
	376	239	23.94	10
Picoxystrobin	145	115	18.72	15

	145	102	18.72	25
Piperophos	320	122	23.94	10
	140	98	23.94	10
Pirimicarb	166	96	14.28	10
	166	71	14.28	30
Pirimiphos-ethyl	333	180	16.92	10
	333	168	16.92	20
Pirimiphos-methyl	290	125	15.72	20
	233	151	15.72	5
Pretilachlor	162	147	19.26	15
	162	132	19.26	20
Probenazole	130	103	15.96	20
	130	77	15.96	30
Prochloraz	180	138	27.54	10
	180	69	27.54	20
Procymidone	96	67	17.91	10
	96	53	17.91	20
Profenofos	208	99	19.35	30
	208	63	19.35	40
Profluralin	317.9	199	13.34	15
	317.9	54.8	13.34	10
Prometon	210	168.1	13.04	5
	210	112	13.04	10
Prometryn	226	184	15.43	10
	199	184	15.43	5
Pronamide(Propyzamide)	175	147	13.60	15
	173	145	13.60	15
Propachlor	176	120	11.60	10
	176	57	11.60	10
Propanil	163	90	14.80	20
	161	126	14.80	25
Propazine	214	172	13.26	15
	214	104	13.26	20
Propetamphos	138	110	13.45	10
	138	64	13.45	15
Propham	179	137	10.16	5
	179	93	10.16	15
Propisochlor	162	144	15.25	15

	162	119	15.25	20
Prothiofos	267	239	19.20	10
	267	221	19.20	20
Pyracarbolid	125.1	55.1	17.01	15
	125	55	17.01	20
Pyraclofos	360	194	26.60	10
	360	97	26.60	30
Pyrazophos	221	193	26.04	10
	221	149	26.04	10
Pyridaben	147	117	27.53	20
	147	91	27.53	40
Pyridalyl	204	176	29.62	10
	204	148	29.62	20
Pyrifenox	171	136	18.43	15
	171	100	18.43	30
Pyrimidifen	184	169	30.31	20
	184	44	30.31	20
Pyriminobac-methyl(E)	302	256	21.93	20
	302	230	21.93	20
Quinalphos	146	118	17.82	15
	146	91	17.82	25
Quinoxyfen	306.8	237	21.93	20
	237	208.1	21.93	30
Quintozene	237	143	13.36	30
	237	119	13.36	30
Secbumeton	196	85	14.00	10
	169	154.1	14.00	5
Silafluofen	286	258	29.86	10
	179	151	29.86	10
Simeconazole	121	101	15.14	10
	121	75	15.14	30
Simetryn	213	185	15.24	10
	213	170	15.24	10
Spiromesifen	272	254	23.27	10
	272	209	23.27	10
Spiroxamine	100	72.1	15.12	5
	100	58.1	15.12	10
Sulfotep	321.9	174	12.24	15

	321.9	146	12.24	25
Sulprofos	156	141	21.43	15
	140	125	21.43	15
TCMTB (Benthiazole)	180	180	19.08	5
	180	136	19.08	20
Tebuconazole	250	125	22.63	30
	250	70	22.63	10
Tebufenpyrad	333	171	24.38	15
	276	171	24.38	10
Tebupirimfos	234	126	14.27	10
	234	110	14.27	10
Tefluthrin	177	127	13.96	20
	177	87	13.96	30
Terbacil	160	116	13.98	5
	160	76	13.98	15
Terbufos	231	175	13.55	10
	231	129	13.55	30
Terbumeton	210	100	13.30	30
	210	68	13.30	40
	210	58	13.30	40
	210	57	13.30	40
	169	154	13.30	10
Terbuthylazine	214	71	13.52	20
	214	43	13.52	40
Terbutryn	241	170	15.81	15
	185	170	15.81	5
Tetrachlorvinphos	330.8	109	18.45	15
	328.8	109	18.45	15
Tetraconazole	336	218	16.59	15
	336	203.8	16.59	30
Tetradifon	159	131	24.78	10
	159	75	24.78	40
Tetramethrin	164	107.1	23.97	10
	164	77.1	23.97	25
Tetrasul	323.9	251.9	21.35	15
	321.7	252	21.35	15
Thiazopyr	349	329	16.10	10
	327	277	16.10	30

Thifluzamide	194	166	19.69	15
	194	125	19.69	25
Thiometon	125	79	12.95	10
	125	47	12.95	15
Tolfenpyrad	383	211.2	33.17	20
	383	171.1	33.17	20
Tolylfluanid	238	137	17.61	20
	238	91	17.61	40
	137	91	17.61	20
	137	65	17.61	40
Tralomethrin	253	174	32.32	5
	253	93	32.32	20
Triadimefon	208	181	16.62	10
	208	99	16.62	30
	168	112	17.93	10
	168	70	17.93	20
	128	65	17.93	25
Tri-allate	268	184	14.22	10
	143	83	14.22	15
Triazophos	161	134	21.40	5
	161	106	21.40	10
Tribufos(Tribuphos)	202	147	19.59	0
	169	57	19.59	5
Triflumizole	206	186	17.97	10
	206	179	17.97	20
Triflumuron	139	111	10.16	15
	139	75	10.16	30
Trifluralin	306	264	12.20	10
	264	160	12.20	15
Uniconazole	234	165	19.50	10
	234	137	19.50	20
Vernolate	128	86	9.34	5
	128	43	9.34	10
Vinclozoline	212	172	15.03	20
	212	145	15.03	30
Zoxamide (decomp)	187	159	23.12	20

Table S3. Identification criteria ion for LC-MS/MS analysis

Pesticide	Precursor ion (*m/z*)	Production ion (*m/z*)	RT (min)	Collision energy (eV)
Aldicarb	207.924	49	2.98	32
Aldicarb	207.924	89	2.98	17
Amisulbrom	465.98	148.06	5.32	50
Amisulbrom	465.98	227	5.32	21
Azamethiphos	325.055	112.119	2.93	37
Azamethiphos	325.055	139.077	2.93	28
Azamethiphos	325.055	183.026	2.93	19
Azoxystrobin	404.12	329.061	3.66	33
Azoxystrobin	404.12	344.076	3.66	27
Azoxystrobin	404.12	372.067	3.66	16
Bendiocarb	224.175	81.284	2.99	34
Bendiocarb	224.175	109.174	2.99	21
Bendiocarb	224.175	167.126	2.99	10
Bensulide	398.02	77.296	4.33	59
Bensulide	398.02	141.104	4.33	35
Bensulide	398.02	158.105	4.33	26
Benzoximate	363.988	105.111	4.86	23
Benzoximate	363.988	183.889	4.86	34
Benzoximate	363.988	198.946	4.86	10
Bixafen	414.132	266	4.39	30
Bixafen	414.132	374	4.39	26
Bixafen	414.132	394	4.39	15
Boscalid	343.04	271.116	3.81	37
Boscalid	343.04	272.091	3.81	34
Boscalid	343.04	307.065	3.81	21
Bromoxynil	275.785	78.833	4.31	30
Bromoxynil	275.785	80.833	4.31	30
Bromoxynil	275.785	193.804	4.31	20
Butocarboxim	213.099	75	2.6	15
Butocarboxim	213.099	156	2.6	8
Carbaryl	202.165	127.152	3.2	31
Carbaryl	202.165	145.11	3.2	11
Carbetamide	236.918	92	2.81	31
Carbetamide	236.918	120	2.81	17

Carbetamide	236.918	192	2.81	5
Chlorantraniliprole	483.985	194	3.5	35
Chlorantraniliprole	483.985	286	3.5	15
Chlorantraniliprole	483.985	453	3.5	18
Chlorimuron-Ethyl	415.075	83.225	3.83	49
Chlorimuron-Ethyl	415.075	121.133	3.83	41
Chlorimuron-Ethyl	415.075	186.013	3.83	21
Chlorobenzuron	306.989	126	4.52	30
Chlorobenzuron	306.989	264	4.52	15
Chlorotoluron	213.08	72.333	3.36	19
Chlorotoluron	213.08	140.071	3.36	26
Chlorotoluron	213.08	167.986	3.36	19
Chromafenozide	395.199	175	4.06	16
Chromafenozide	395.199	339	4.06	5
Cinosulfuron	414.11	121.156	2.88	27
Cinosulfuron	414.11	157.149	2.88	24
Cinosulfuron	414.11	183.098	2.88	20
Crufomate	292.1	204	6.37	26
Crufomate	292.1	236	6.37	18
Cyazofamid	325	108.169	4.2	17
Cyazofamid	325	216.088	4.2	19
Cyazofamid	325	217.067	4.2	21
Cycloprothrin	498.881	181	6.48	33
Cycloprothrin	498.881	208	6.48	15
Cymoxanil	199.05	111.02	2.32	20
Cymoxanil	199.05	128.02	2.32	10
Dicrotophos	237.906	72	1.79	29
Dicrotophos	237.906	127	1.79	17
Dicrotophos	237.906	193	1.79	7
Dimethomorph_E	388.155	165.118	3.89	34
Dimethomorph_E	388.155	301.105	3.89	23
Dimethomorph_Z	388.155	165.118	3.73	34
Dimethomorph_Z	388.155	301.105	3.73	23
Ethaboxam	321.098	183.058	3.2	23
Ethaboxam	321.098	200.04	3.2	26
Ethaboxam	321.098	237.071	3.2	19
Ethametsulfuron_methyl	411.134	83	3.21	52
Ethametsulfuron_methyl	411.134	168	3.21	32

Ethametsulfuron_methyl	411.134	196	3.21	18
Ethiofencarb	226.165	77.279	3.19	45
Ethiofencarb	226.165	107.183	3.19	18
Fenhexamid	302.12	55.424	4.03	38
Fenhexamid	302.12	97.255	4.03	26
Fenhexamid	302.12	143.089	4.03	39
Fenpyroximate	422.235	214.136	6.55	32
Fenpyroximate	422.235	231.115	6.55	26
Fenpyroximate	422.235	366.126	6.55	16
Ferimzone	255.1	117.1	3.43	26
Ferimzone	255.1	124.1	3.43	21
Ferimzone	255.1	132.1	3.43	21
Fluacrypyrim	427.15	115.07	5.02	59
Fluacrypyrim	427.15	145.07	5.02	27
Fluazinam	462.828	387	5.74	19
Fluazinam	462.828	398	5.74	20
Fluazinam	462.828	416	5.74	23
Flubendiamide	681	254	4.36	27
Flubendiamide	681	271.9	4.36	17
Flubendiamide	681	274	4.36	16
Flufenacet	364.068	124.111	4.11	32
Flufenacet	364.068	152.04	4.11	19
Flufenacet	364.068	194.04	4.11	10
Flufenoxuron	488.939	113.111	6.29	55
Flufenoxuron	488.939	141	6.29	39
Flufenoxuron	488.939	158	6.29	19
Fluometuron	232.911	145	3.26	34
Fluometuron	232.911	160	3.26	27
Fluometuron	232.911	168	3.26	23
Fluquinconazole	376	108.149	4.06	27
Fluquinconazole	376	349.076	4.06	19
Fluridone	330.115	91	3.61	48
Fluridone	330.115	115	3.61	55
Fluridone	330.115	310	3.61	25
Flusulfamide	412.985	171	4.84	43
Flusulfamide	412.985	179	4.84	48
Flusulfamide	412.985	283	4.84	37
Forchlorfenuron	248.035	93.236	3.43	36

Forchlorfenuron	248.035	129.127	3.43	20
Forchlorfenuron	248.035	155.062	3.43	18
Hexaflumuron	458.92	174.866	5.29	37
Hexaflumuron	458.92	300.924	5.29	22
Hexaflumuron	458.92	438.932	5.29	14
Hexazinone	253.167	71.1	3.08	32
Hexazinone	253.167	171	3.08	16
Hexythiazox	353.132	168	6.25	24
Hexythiazox	353.132	228	6.25	14
Imazamox	306.151	246	2.41	23
Imazamox	306.151	261	2.41	19
Imazamox	306.151	264	2.41	16
Imazapic	276.139	145	2.48	36
Imazapic	276.139	163	2.48	25
Imazapic	276.139	231	2.48	19
Imazaquin	312.138	198	3.07	28
Imazaquin	312.138	199	3.07	29
Imazethapyr	290.225	177.108	2.84	30
Imazethapyr	290.225	230.081	2.84	26
Imazethapyr	290.225	245.121	2.84	23
Imibenconazole	411.035	125.13	5.77	30
Imibenconazole	411.035	296.003	5.77	23
Imibenconazole	411.035	342.027	5.77	18
Imidacloprid	256.099	175	1.9	20
Imidacloprid	256.099	209	1.9	17
Ipconazole	333.933	89	5.19	62
Ipconazole	333.933	125	5.19	49
Isoprocarb	194.195	77.26	3.36	37
Isoprocarb	194.195	95.22	3.36	18
Isoproturon	207.225	72.312	3.45	21
Isoproturon	207.225	134.182	3.45	26
Isoxaben	332.927	107	3.81	54
Isoxaben	332.927	150	3.81	36
Isoxaben	332.927	165	3.81	19
Lenacil	234.931	135	3.38	31
Lenacil	234.931	136	3.38	28
Lenacil	234.931	153	3.38	16
Lufenuron	508.9	174.9	5.97	37

Lufenuron	508.9	325.8	5.97	17
Lufenuron	508.9	339	5.97	10
Malaoxon	314.89	99	3.05	23
Malaoxon	314.89	127	3.05	12
Malaoxon	314.89	269	3.05	5
Mepanipyrim	224.195	77.278	4.26	39
Mepanipyrim	224.195	106.194	4.26	29
Mepanipyrim	224.195	209.106	4.26	25
Mephosfolan	270	140	4.67	25
Mephosfolan	270	168.071	4.67	17
Metamifop	441.136	123.006	5.51	29
Metamifop	441.136	180.031	5.51	19
Metamifop	441.136	288.025	5.51	16
Metamitron	202.91	104	2.07	26
Metamitron	202.91	174	2.07	16
Metamitron	202.91	175	2.07	15
Methabenzthiazuron	222.145	124.145	3.53	32
Methabenzthiazuron	222.145	150.104	3.53	36
Methabenzthiazuron	222.145	165.105	3.53	19
Methiocarb	226	121.045	3.76	36
Methiocarb	226	169.082	3.76	10
Methomyl	163	73	0.8	29
Methomyl	163	88	0.8	10
Methomyl	163	106	0.8	10
Methoxyfenozide	369.27	91.232	3.89	47
Methoxyfenozide	369.27	133.131	3.89	25
Methoxyfenozide	369.27	149.114	3.89	20
Metolcarb	166.098	91	2.59	23
Metolcarb	166.098	94	2.59	28
Metominostrobin	285.149	194	3.48	22
Metominostrobin	285.149	196	3.48	17
Metominostrobin	285.149	238	3.48	12
Neburon	275.062	88.143	6.42	16
Neburon	275.062	114.179	6.42	14
Nitenpyram	271.15	126.097	0.71	30
Nitenpyram	271.15	224.099	0.71	18
Nitenpyram	271.15	237.098	0.71	21
Noruron	223.238	67.107	5.67	29

Noruron	223.238	71.964	5.67	27
Novaluron	490.985	304.961	5.39	19
Novaluron	490.985	332.96	5.39	22
Novaluron	490.985	470.974	5.39	14
Oxaziclomefone	376.075	161.057	5.44	28
Oxaziclomefone	376.075	190.1	5.44	16
Phenmedipham	301.148	93	3.5	36
Phenmedipham	301.148	108	3.5	27
Phenmedipham	301.148	136	3.5	19
Pinoxaden	400.949	101	4.86	37
Pinoxaden	400.949	159	4.86	41
Pinoxaden	400.949	317	4.86	19
Promecarb	208	109.25	3.84	16
Promecarb	208	151.2	3.84	9
Propaquizafop	443.902	299	5.54	25
Propaquizafop	443.902	327	5.54	28
Propaquizafop	443.902	371	5.54	17
Propoxur	210.185	65.374	2.99	38
Propoxur	210.185	93.215	2.99	27
Propoxur	210.185	111.177	2.99	17
Prosulfocarb	251.931	65	5.35	51
Prosulfocarb	251.931	91	5.35	30
Prosulfocarb	251.931	128	5.35	11
Prothioconazole	341.862	100	4.62	26
Prothioconazole	341.862	264	4.62	21
Prothioconazole	341.862	306	4.62	18
Pyraclonil	315.1	169.125	4.95	29
Pyraclonil	315.1	276.125	4.95	16
Pyraclostrobin	388.15	133.126	4.8	35
Pyraclostrobin	388.15	149.093	4.8	31
Pyraclostrobin	388.15	163.107	4.8	26
Pyraflufen-ethyl	412.884	253	4.43	31
Pyraflufen-ethyl	412.884	261	4.43	27
Pyraflufen-ethyl	412.884	339	4.43	17
Pyrazolate	439.049	91.222	4.96	33
Pyrazolate	439.049	155.071	4.96	21
Pyrazolate	439.049	172.929	4.96	20
Pyribenzoxim	610.208	180.04	5.58	10

Pyribenzoxim	610.208	381.071	5.58	34
Pyribenzoxim	610.208	413.04	5.58	10
Pyribenzoxim	610.181	180	5.82	33
Pyribenzoxim	610.181	413	5.82	13
Pyributicarb	331.2	108.207	5.84	30
Pyributicarb	331.2	133.19	5.84	27
Pyributicarb	331.2	162.2	5.84	28
Pyridate	379.136	104	7.43	36
Pyridate	379.136	207	7.43	17
Pyridate	379.136	351	7.43	5
Pyrimethanil	200.195	143.121	3.61	28
Pyrimethanil	200.195	168.102	3.61	33
Pyrimethanil	200.195	181.14	3.61	40
Pyriproxyfen	322.19	78.275	5.87	59
Pyriproxyfen	322.19	96.237	5.87	19
Pyriproxyfen	322.19	185.104	5.87	25
Pyroquilon	174.19	117.171	2.94	36
Pyroquilon	174.19	130.17	2.94	40
Pyroquilon	174.19	132.156	2.94	26
Quinoclamine	208.032	77	2.98	36
Quinoclamine	208.032	105	2.98	24
Quinoclamine	208.032	208	2.98	10
Rimsulfuron	432.1	182.2	3.15	21
Rimsulfuron	432.1	325.2	3.15	17
Simazine	202.126	104	3.08	24
Simazine	202.126	124	3.08	17
Simazine	202.126	132	3.08	18
Spirodiclofen	411.03	71.333	6.45	15
Spirodiclofen	411.03	212.817	6.45	35
Spirodiclofen	411.03	312.889	6.45	10
Sulfentrazone	404.068	178	3.07	30
Sulfentrazone	404.068	307	3.07	34
Sulfentrazone	404.068	387	3.07	18
Tebufenozide	353.183	105.183	4.36	38
Tebufenozide	353.183	133.111	4.36	18
Tebufenozide	353.183	297	4.36	10
Tebuthiuron	228.913	116	3.09	27
Tebuthiuron	228.913	157	3.09	26

Tebuthiuron	228.913	172	3.09	16
Teflubenzuron	378.856	196	5.78	23
Teflubenzuron	378.856	339	5.78	15
Teflubenzuron	378.856	359	5.78	10
Tepraloxydim	342.112	166.125	5.91	22
Tepraloxydim	342.112	250.196	5.91	13
Thenylchlor	324.068	99.183	4.14	31
Thenylchlor	324.068	127.111	4.14	10
Thiamethoxam	292.077	132	1	22
Thiamethoxam	292.077	181	1	20
Thiodicarb	355.1	88.07	3.29	19
Thiodicarb	355.1	108.07	3.29	17
Tiadinil	265.909	71.222	3.98	15
Tiadinil	265.909	178	3.98	16
Tiadinil	265.909	237.96	3.98	10
Tribenuron-methyl	395.996	155.035	3.37	14
Tribenuron-methyl	395.996	181.012	3.37	19
Tribenuron-methyl	395.996	198.952	3.37	16
Tricyclazole	190.1	109.1	2.85	36
Tricyclazole	190.1	136	2.85	29
Tricyclazole	190.1	163	2.85	24
Tridemorph	297.991	98	4.86	29
Tridemorph	297.991	116	4.86	24
Tridemorph	297.991	130	4.86	25
Trifloxystrobin	409.14	116.173	5.15	23
Trifloxystrobin	409.14	145.085	5.15	44
Trifloxystrobin	409.14	186.086	5.15	20
235-Trimethacarb	194.113	107	3.44	36
235-Trimethacarb	194.113	122	3.44	30
235-Trimethacarb	194.113	137	3.44	12
Triticonazole	317.91	70	4.11	32
Triticonazole	317.91	125	4.11	31
XMC	179.922	77	5.57	42
XMC	179.922	123	5.57	9
